# The multifaceted nature of IL-10: regulation, role in immunological homeostasis and its relevance to cancer, COVID-19 and post-COVID conditions

**DOI:** 10.3389/fimmu.2023.1161067

**Published:** 2023-06-08

**Authors:** Valentina Carlini, Douglas M. Noonan, Eslam Abdalalem, Delia Goletti, Clementina Sansone, Luana Calabrone, Adriana Albini

**Affiliations:** ^1^ Istituto di Ricovero e Cura a Carattere Scientifico (IRCCS), MultiMedica, Milan, Italy; ^2^ Department of Biotechnology and Life Sciences, University of Insubria, Varese, Italy; ^3^ Translational Research Unit, National Institute for Infectious Diseases Lazzaro Spallanzani- Istituto di Ricovero e Cura a Carattere Scientifico (IRCCS), Rome, Italy; ^4^ Stazione Zoologica Anton Dohrn, Istituto Nazionale di Biologia, Ecologia e Biotecnologie Marine, Napoli, Italy; ^5^ Istituto di Ricovero e Cura a Carattere Scientifico (IRCCS) European Institute of Oncology IEO-, Milan, Italy

**Keywords:** IL-10, STAT3, cytokine storm, alarmin, COVID - 19, Post-COVID

## Abstract

Interleukin-10 (IL-10) is a pleiotropic cytokine that has a fundamental role in modulating inflammation and in maintaining cell homeostasis. It primarily acts as an anti-inflammatory cytokine, protecting the body from an uncontrolled immune response, mostly through the Jak1/Tyk2 and STAT3 signaling pathway. On the other hand, IL-10 can also have immunostimulating functions under certain conditions. Given the pivotal role of IL-10 in immune modulation, this cytokine could have relevant implications in pathologies characterized by hyperinflammatory state, such as cancer, or infectious diseases as in the case of COVID-19 and Post-COVID-19 syndrome. Recent evidence proposed IL-10 as a predictor of severity and mortality for patients with acute or post-acute SARS-CoV-2 infection. In this context, IL-10 can act as an endogenous danger signal, released by tissues undergoing damage in an attempt to protect the organism from harmful hyperinflammation. Pharmacological strategies aimed to potentiate or restore IL-10 immunomodulatory action may represent novel promising avenues to counteract cytokine storm arising from hyperinflammation and effectively mitigate severe complications. Natural bioactive compounds, derived from terrestrial or marine photosynthetic organisms and able to increase IL-10 expression, could represent a useful prevention strategy to curb inflammation through IL-10 elevation and will be discussed here. However, the multifaceted nature of IL-10 has to be taken into account in the attempts to modulate its levels.

## Introduction

Cytokines are a broad category of soluble proteins or glycoproteins with low molecular weight (ranging from 6 to 70 kDa) that are produced transiently, in response to various biological stimuli, by nearly every cell type and affecting virtually all main cellular processes (1–3). These molecules are crucial to orchestrate cell-to-cell communication and biological functions (1–3). They can act locally, either via autocrine and paracrine signaling, respectively on the same cells that produce them or on cells close to the site of release. Several cytokines are also capable of acting systemically, via endocrine signaling, on cells located in other body districts reached through the blood or lymphatic stream (4). Cytokines act by binding to specific transmembrane and membrane-anchored receptors located on the target cells and activating downstream intracellular signaling cascades that usually result in gene expression modulation (5, 6). Some cytokine receptors also exist in soluble form and can function as either antagonists or agonists of cytokine signaling, forming decoy receptors or functional receptors, respectively (2, 6). Their activity is highly specific: the expression pattern of cytokine receptors is unique for every tissue and varies among different cell types, determining to which cytokine a particular cell/tissue will respond to (6). Cytokines also establish complex networks with each other, where one cytokine can potentiate/contrast the action or stimulate/inhibit the production of other cytokines (2).

Cytokines play a pivotal role in the regulation of many physiological processes, including cytoskeletal organization, stem cell differentiation, embryonic development, cell proliferation, activation, migration, wound healing, survival and apoptosis (6–9). They are also key regulators of the innate and adaptive immunity, coordinating humoral, cytotoxic and cellular immune response, mediating communication between immune and non-immune cells, controlling immune cell trafficking and tissue organization, affecting microenvironment and regulating inflammation (1, 10–12). Given the high pleiotropic activity of these molecules, cytokines are present at very low or undetectable concentrations in body fluids and tissues under homeostatic conditions, but, when required by the physiopathological context, they can rapidly increase up to 1000 fold (13).

Since cytokines have such a fundamental influence on immune system and body’s health, any dysregulation in their secretion or signaling, and they are critically implicated in the genesis and/or progression of several human pathological conditions, among which, cancer, infectious and immune diseases (1, 13, 14) they represent important biomarkers and targets.

A dysregulation of the immune system is a characteristic of the COronaVIrus Disease 2019 (COVID-19) global pandemic, caused by the severe acute respiratory syndrome coronavirus 2 (SARS-CoV-2) (15–17). A growing body of clinical data suggests that the more severe and lethal forms of COVID-19 syndrome are associated with self-feeding massive release of pro-inflammatory cytokines, defined “cytokine storm” (18–20), which triggers and sustains a systemic hyper-activated inflammatory response that finally leads to acute respiratory distress syndrome (ARDS), multi-organ failure and even death (17, 21).

This COVID-19 pandemic has posed huge challenges to the health care system worldwide due to its widespread diffusion, the severity of clinical picture (17), and the numerous variants, with more than 765 million confirmed cases and over 6.9 million deaths reported globally (22). The rapid availability of vaccines has brought an enormous progress. Unfortunately, in case of infection, clinical interventions capable of effectively treating COVID-19 patients entering the cytokine storm phase have demonstrated a highly variable margin of success depending on timing and patient selection (23). In addition, a considerable percentage of patients who had mild or severe disease do not fully recover but continue to manifest a range of persistent debilitating multi-organ symptoms for weeks or even months after the acute infection. These conditions have been reported in literature as “long COVID”, “post-COVID-19 syndrome”, “post-acute sequelae of COVID-19 (PACS)” or “post-acute COVID-19 syndrome” and they still leave many open questions about the pathogenetic mechanisms and potential therapeutic approaches (24–28). Although the vaccines have brought substantial benefits to halt the COVID-19 pandemic, both reducing rate of SARS-CoV-2 new infection and decreasing mortality and risk of serious complications, there is actually no proven effective treatment against post-COVID-19 and the real impact of vaccines on patients who have this syndrome remains still unclear (28–30). Therefore, a thorough understanding of the molecular mechanisms underlying COVID-19 and post-COVID-19 as well as the design of novel targeted therapeutic interventions remains a priority for biomedical research.

In light of the importance demonstrated by the “cytokine storm” in driving the immunopathological process of COVID-19, numerous therapeutic strategies capable to prevent/reduce the over-production of pro-inflammatory cytokines have been proposed to suppress/attenuate COVID-19 hyper-inflammation and ameliorate its severe complications (31–33). These pharmacological agents comprise non-specific immune modulators, such as corticosteroids, hydroxychloroquine, interferons, cardiovascular drugs, such as statins and renin–angiotensin–aldosterone system (RAAS) inhibitors, and specific immune modulators, such as Janus kinase (Jak) inhibitors, humanized anti-interleukin-6 (IL-6), anti-IL-1 receptor and anti-tumor necrosis factor alpha (TNF-α) monoclonal antibodies (34–41). Very likely, they can be valid therapeutic options also for managing post-COVID-19 complications (42).

In this scenario, a novel candidate that appears to be particularly worth to be exploited as effective anti-inflammatory molecule is IL-10, a cytokine that has gained increasing interest from clinical medicine in different therapeutic settings due to its potent immunomodulatory properties on a broad spectrum of cells (43–45).

Recent evidence has outlined IL-10 as associated with severity and mortality for patients with acute or post-acute SARS-CoV-2 infection (46). IL-10 can act like an endogenous “danger signal” released in response to the peak of circulating pro-inflammatory cytokines and having the purpose to protect the organism from damage caused by harmful hyperinflammatory state (43, 47).

## IL-10 structure and signaling pathway

Human IL-10 is encoded by the IL-10 gene, located on the long arm of chromosome 1 (48). The IL-10 gene promoter is characterized by the presence of positive and negative regulatory sequences as well as polymorphisms that can significantly affect IL-10 expression between individuals (49, 50). IL-10 is a member of the class II cytokine family and its biologically active form is a soluble 36 kDa homodimer, comprising two monomers with six α-helices structure and stabilized by two intrachain disulfide bonds (51). The cellular response to IL-10 starts with the binding of an IL-10 homodimer to a heterotetrametric IL-10 receptor (IL-10R) complex, belonging to the interferon receptor family and comprised of two ligand-binding IL-10R-alpha (IL-10RA) subunits and two accessory signal-transducing IL-10R-beta (IL-10RB) subunits (52, 53). IL-10RA is the main responsible for directing ligand and target specificity: it recognizes IL-10 with high-affinity (50) and it is mainly expressed by lymphocytes, macrophages and dendritic cells at basal level, but can be upregulated by various cells upon their activation (54). Instead, IL-10RB has lower affinity or no direct binding to IL-10, is constitutively expressed by nearly all cell types and is shared by the receptor complex of other IL-10 family cytokines, such as IL-22 and IL-26 (50, 55). The signaling cascade in IL-10 responding cells is mediated by theJak1/Tyrosine kinase 2 (Tyk2)/signal transducer and activator of transcription 3 (STAT3) pathway. The binding of IL-10 homodimer to the IL-10RA extracellular domain leads to its oligomerization with the IL-10RB and the following phosphorylation of the enzymes Jak1 and Tyk2, associated with the intracellular domain of alpha and beta subunits, respectively. Upon their phosphorylation, these kinases further phosphorylate two functional tyrosine motifs on the intracellular domain of the IL-10RA. This allows the recruitment of STAT3 and its subsequent phosphorylation by Jak1 and Tyk2 (43, 50). Once phosphorylated, STAT3 dimerizes and translocates into the nucleus, where it binds to STAT-consensus elements of target gene promoters and initiates their transcriptional program (43, 50, 56). One of the actions of STAT3-responsive genes is the suppression of cytokine signaling 3 (SOCS-3), which inhibits mitogen-activated protein kinase (MAPK) activation, NF-κB nuclear translocation, and the resulting expression of pro-inflammatory genes. It also functions as a negative feedback regulator of IL-10 signaling, by inhibiting Jak1 and consequently the Jak1/Tyk2/STAT3 pathway. Another element induced by STAT3 is the IL-1 receptor antagonist (IL-1RN), a decoy protein that, binding to IL-1 receptor, prevents the interaction of IL-1β with its receptor and the following activation of pro-inflammatory signaling. Moreover, STAT3 suppresses STAT6 activation and consequently inhibits the expression of IL-4/IL-13-responsive genes in monocytes and dendritic cells (DCs) (50, 57–59).

In addition to STAT3, IL-10RA may simultaneously phosphorylate and activate STAT1 and STAT5 in monocytes and regulatory T (Treg) cells. By this action, it leads to the formation of different STAT heterocomplexes and to the subsequent generation of multiple downstream transcriptional effects (60). Furthermore, additionally to Jak1/Tyk2/STAT3 pathway, IL-10 may also modulate transcription by the activation of PI3K/Akt/Glycogen Synthase Kinase 3 Beta (GSK3β) and PI3K/Akt/mTORC1 signaling cascades in macrophages (43).

There is evidence of IL-10 and IL-10R deficiencies which are monogenic inborn errors of immunity (IEI) causing early-onset inflammatory bowel diseases (IBD) (61, 62). Consanguinity is reported in all evaluable patients with IL-10 deficiency and in 38% of patients with IL-10R deficiency (23% of patients with IL-10RA, and 79% of patients with IL-10RB deficiency). The common associated pathologies are auto-inflammation and enteropathy. Dermatological manifestations as well as lymphoma not Epstein Barr Virus (EBV)-related, and failure to thrive are associated with IL-10R deficiency (63, 64).

## IL-10 cellular sources

When originally described by Fiorentino and colleagues in 1989, IL-10 was classified as a cytokine specifically secreted by T helper 2 (Th2) cells (65), however, it was subsequently widely recognized that it can be produced by many myeloid and lymphoid cells (50). Among these, CD4+ Th1, Th2 and Th17 cells, and Treg cells, DCs, monocytes and macrophages are main producers of IL-10 (43, 50). Recently, microglia and cardiac macrophages have been also identified as producers of IL-10 (66, 67).

In CD4+ Th cells, IL-10 production occurs downstream of T cell receptor (TCR) activation and the subsequent activation of Ras, ERK1/2 and transcription factor AP1 (43). In Th2 cells, IL-10 synthesis is induced by IL-4/STAT6 signaling and requires GATA binding protein 3 (GATA3) transcription factor (68). Th1 and Th17 can secrete IL-10 under the correct set of conditions. In Th1 cells, IL-10 production requires STAT4, strong TCR activation (i. e. increased expression of Delta-like-4 ligand and inducible T cell co-stimulator ligand (ICOSL) on DCs) and IL-12 (69).

In Th17 cells, IL-10 expression is induced by the cytokines IL-6 (70), IL-24 (71) and IL-27 (72), and it is mediated by STAT3 and, in some cases, STAT-1 signaling (73). Several transcription factors are involved in regulating IL-10 production in T cells, including Blimp-1, cMaf, AhR, Bhlhe40 (43, 50).

Natural and induced FoxP3+ Treg cells can secrete IL-10 in a STAT3 dependent manner and use it to control immune responses against self-antigens at the environmental interfaces (74). FoxP3- Treg cells secrete IL-10 following differentiation from naive CD4+ T cells under various stimuli, including cytokines, such as interferon gamma (INF-γ), immunosuppressive drugs, stimulation with soluble antigens or immature DCs and co-stimulation with CD2, CD46 or ICOSL (75).

In macrophages and DCs, IL-10 expression is regulated by cytokines, such as type I IFN, and by the activation, downstream of Toll-like receptor (TLR) signaling, of ERK1/2, p38, NF-κB and phosphoinositide-3-kinase (PI3K) serine/threonine protein kinase B (Akt) pathways (43, 50, 76).

In addition to CD4+ T cells, DCs and macrophages, most adaptive immunity cell types, including CD8+ cytotoxic T cells and B cells, as well as various innate immunity cell types, including mast cells, natural killer (NK) cells, and eosinophils can also be sources of IL-10 in particular contexts (77).

CD8+ T‐cells become significant producers of IL-10 during hypoxia and viral infection (78), in response to TCR activation, IL-21 stimulation or interaction with CD40 ligand on activated pDCs (75). Mast cells directly induce IL-10 expression following Toll-like receptor 4 (TLR4) activation by lipopolysaccharide (LPS) or during allergic dermatitis or skin damage (75). In B cells, IL-10 production occurs following stimulation with autoantigens, ligands of TLR4 and TLR9, or vitamin D3 (79), while NK cells release IL-10 during systemic infection (80). However, unlike other myeloid cells and in contrast to mouse neutrophils, human neutrophils are unable to produce or secrete IL-10, also after stimulation with bacterial and inflammatory molecules such as serum amyloid or LPS (81). Cassatella’s and Bazzoni’s labs showed that IL-10 induced transcriptional repression of CXCL8 and TNF-α genes in human monocytes pretreated with LPS (82). The inhibitory effect of IL-10 on cytokine transcription consists of two distinct sequential phases: an early phase, occurring rapidly and in a protein synthesis-independent manner, followed by a second delayed phase, that occurs after 60 minutes and is dependent on protein synthesis (82).

In addition, some non-immune cell types, including intestinal epithelial cells, intestinal fibroblasts and skin keratinocytes, produce IL-10 in response to certain stimuli, comprising infection, UV radiation, tissue injury and damage (83–86), and even different tumor cells, such as melanoma, breast and colon carcinoma cells, have demonstrated IL-10 secretion ability (87–90).

## IL-10 systemic effects

IL-10 was initially defined as “cytokine synthesis inhibitory factor” due to its inhibitory activity on IL-2 and interferon-γ (IFN-γ) release by Th1 cells (65), however it is now commonly considered as a key immunoregulatory cytokine with pleiotropic activities, exerting multiple and sometimes even opposite effects on immune cells.

IL-10 functions as a double-edged sword on the immune system: on one hand it has emerged as a potent anti-inflammatory and immunosuppressive cytokine, on the other hand it can also have immunostimulatory properties (50, 91, 92). The different sources of IL-10 and the type of target cells on which it acts, as well as the site and timing of its secretion are critical features to activate multiple signal transduction pathways, each one contributing to different functions towards the inhibition or the activation of immune cells (79).

IL-10 is a master regulator of immunity during infection with viruses, bacteria, fungi, protozoa and other pathogens, playing a key, and often essential, role in limiting or terminating inflammation and in the consequent host protection. IL-10 production by innate immune cells generally occurs later compared to that of pro-inflammatory cytokines released in the early phase of the inflammatory process. IL-10 secreted at the site of ongoing inflammation is responsible for maintaining the right balance between effective pathogen elimination and prevention of detrimental immune-mediated response against infections, resulting in the restoration of normal tissue homeostasis (47, 79, 93, 94). At the same time, numerous pathogens induce IL-10 up-regulation during the infection and exploit the immunosuppressive activity of this cytokine to escape host immune system and promote a microenvironment that favors their tolerance and long-term survival (79).

IL-10 exerts strong immunosuppressive effects on monocytes, macrophages, which are the cells with the higher expression of IL-10R, and dendritic cells (50). It inhibits the ability of these cells to produce pro-inflammatory cytokines (including IL-1α and β, IL-6, IL-12, IL-18, and TNF-α) and chemokines (CCL2, CCL12, CCL5, IL-8, CXCL10, and CXCL2) and prevents their differentiation, maturation and migration to lymphoid organs (50). It also suppresses the antigen-presenting capabilities to Th1 and Th2 of monocytes and APCs by down-regulating their expression of the class II major histocompatibility complex (MHC II) (95) and the co-stimulatory molecules CD54 (intercellular adhesion molecule-1, ICAM-1), CD80 and CD56 (96–99). Moreover, it can act on CD4+ T cells by inhibiting their antigen-specific activation and proliferation in lymph nodes, limiting their secretion of cytokines, such as IL-2, IFN-γ, IL-4, IL-5 and TNF-α, and their cytotoxic activity (45, 100, 101) and inducing their long-term anergy through the block of CD28 co-stimulatory signaling (102, 103). Therefore, through these coordinated actions, IL-10 leads to the shutdown of the inflammatory immune response, both directly, by the suppression of macrophages and dendritic cells activity, and indirectly, by limiting T cells activation, differentiation and effector function and promoting peripheral tolerance (43, 96).

On the other hand, IL-10 exhibits several immunostimulatory activities. This cytokine is a potent stimulator of B lymphocytes: it prevents apoptosis in germinal cells, enhances cell growth, proliferation and activation and drives differentiation into immunoglobulin-secreting plasma cells (15, 92, 104, 105). IL-10 plays also an important role in differentiation and functioning of the Tregs (106, 107) and promotes the survival of T cells otherwise destined to apoptotic cell death (108, 109). Regulatory B cells (Bregs), representing B cells immune-suppressive fractions, regulate inflammation primarily through an interleukin 10 mediated inhibitory mechanism (110, 111). In addition, IL-10 induces thymocytes proliferation, by upregulating the expression of CD3 and CD8 molecules (112). It also enhances the production of IFN-γ and granzyme, improves MHC expression and facilitates antigen recognition, promoting in this way the survival, expansion and cytotoxic activity of antigen activated CD8+ T cells. IL-10 is critically involved in the generation and/or sustaining of effector CD8+ memory T cells too (112).

IL-10 promotes NK cell proliferation and migration and enhances their cytolytic activity and effector functions (113–116). Furthermore, IL-10 directly stimulates mast cells, enhancing their expansion, survival, and activation, upregulating their expression of high-affinity IgE receptors (FcϵRI) and increasing their production of pro-inflammatory cytokines (117).

On murine T cells, IL-10 can function as growth cofactor, stimulating a strong proliferative response of thymocytes in presence of IL-2 and IL-4 (118), and as cytotoxic differentiation factor, promoting IL-2-driven proliferation and differentiation of precursor CD8+ splenocytes into effector CTL (119). IL-10 reveals powerful immunostimulatory properties *in vivo* as well: infusion of exogenous IL-10 in mice recipients of fully allogeneic donor grafts leads to increased graft rejection and graft-versus-host-disease (GVHD)-induced mortality (120). In transgenic murine models, IL-10 expression in the islets of Langerhans results in marked pancreatic inflammation and pronounced recruitment of macrophages, T and B lymphocytes to the pancreas (121). Furthermore, local production of IL-10 by islet cells induces an early development and increased prevalence of autoimmune diabetes in non-obese diabetic mice and accelerates immune-mediated destruction of beta cells (122, 123).

In addition to its broad range activity on the immune system, IL-10 also exerts critical actions on non-immune cells. IL-10 has a fundamental role in central and peripheral nervous system homeostasis, reducing neuronal injury during infection, inflammation, ischemia and trauma, and increasing neuron survival and axon regeneration as well as modulating adult neurogenesis (43, 66, 124). Furthermore, IL-10 is an important regulator of epithelial wound repair and plays a key function in gut homeostasis, promoting wound closure and stimulating intestinal epithelial cell proliferation (43). In dermal wounds, IL-10 promotes regenerative tissue repair via STAT3-dependent regulation of fibroblast-specific hyaluronan synthesis, recruits endothelial progenitor cells (EPCs) and leads to increased vascular structures and faster re-epithelialization (125).

## Regulation of IL-10 production and its double role in immunological homeostasis

IL-10 plays a fundamental role in maintaining host homeostasis at both local and global level, ensuring the fine equilibrium between pro- and anti-inflammatory immune response required to achieve an effective clearance of infecting pathogens and preventing, at the same time, tissue damage occurrence (50, 79). Therefore, in physiological conditions IL-10 production is under a highly dynamic and finely balanced modulation to orchestrate the different immunological activities in a cell-specific manner and to control the inflammatory response force and duration. Several transcription factors, expressed and activated by both distinct and overlapping signaling pathways, are involved in the positive or negative modulation of IL-10 transcription. In addition, a number of common and cell-specific regulatory molecular mechanisms act at epigenetic, post-transcriptional, translational, and secretory level to silence or improve IL-10 expression in the immune effector cells and to ensure the appropriate secretion of the cytokine (79, 126–129). IL-10 expression by immune cells can be regulated, in response to bacterial toxin such as LPS and other environmental stimuli, by alterations in cell metabolic profile, or by accumulation of certain metabolites (43). Consequently, the cells that are main producers and targets of IL-10 as well as the pattern of spatial distribution and temporal availability of this cytokine, may specifically differ between tissues and even in the same tissue, depending on the particular host’s immune status (79). Given its fundamental immunoregulatory properties, IL-10 can equally promote the propagation or the shutdown of inflammatory responses and also direct the fate of parasite, bacterial and viral infections (43).

**Figure 1:**
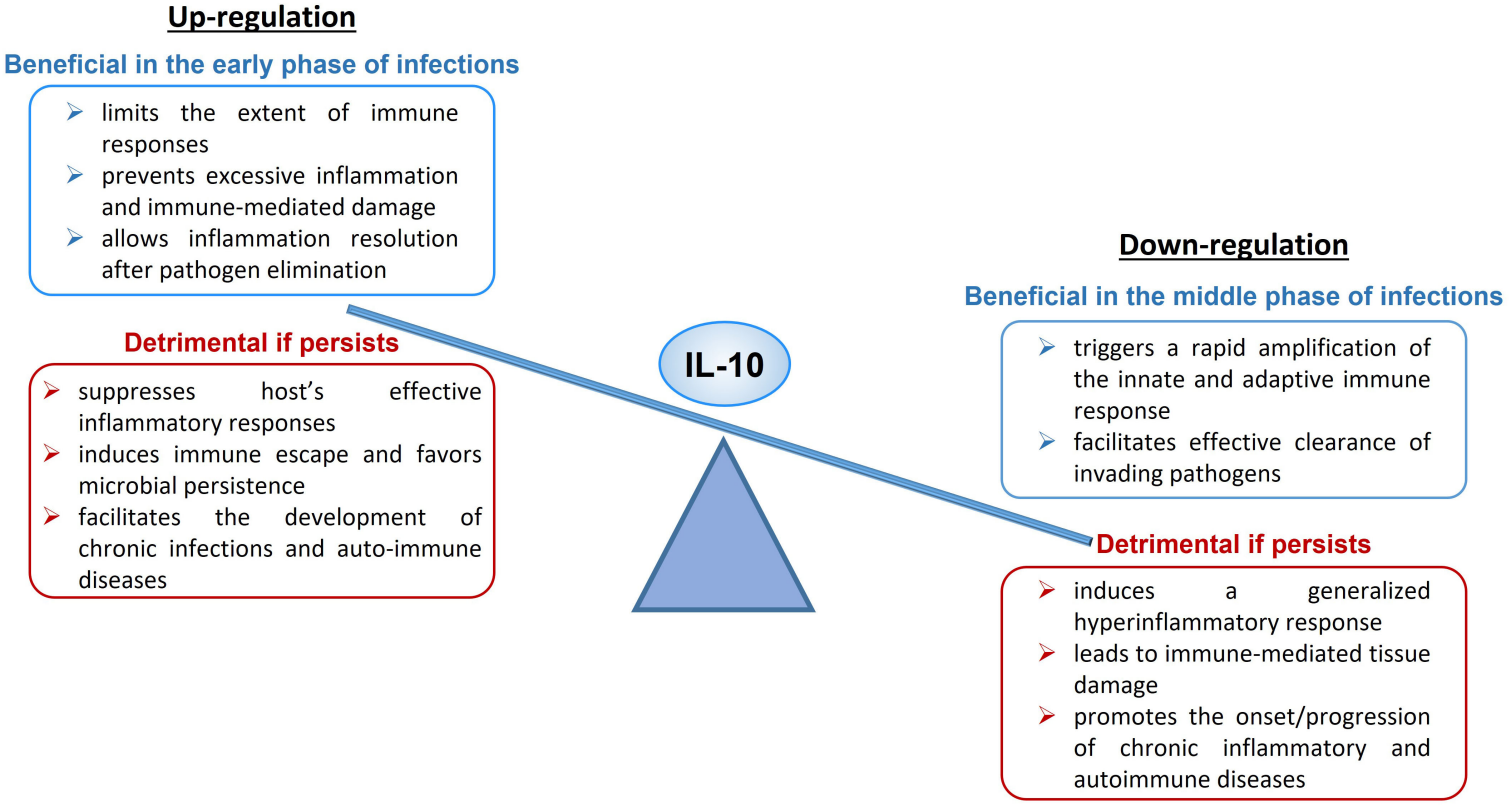
Up- and Down-regulation of IL-10, beneficial and detrimental effects.

Upregulation of IL-10 expression or enhanced signaling can have both a protective and a harmful effect on the organism (**Figure 1**). During acute infections, IL-10 limits the magnitude of the immune responses, preventing excessive inflammation and protecting tissues from immune-mediated damage, and allows inflammation resolution when the pathogen is cleared (47). An excess of IL-10 production or signaling can suppress host’s effective inflammatory responses, induce tolerance and immune escape and favor microbial persistence, leading to the establishment of chronic or latent infections (47, 79) and facilitating the development of auto-immune diseases. As example, it has been demonstrated that IL-10 production is crucial to counter-regulate the harmful inflammatory response activated during acute infections with T. gondii (130), T. cruzi (131), H. hepaticus (132, 133) and influenza (134), while increased IL-10 expression level has been linked to reduced T cell activity and enhanced pathogen replication during chronic infections with T. gondii (130, 135), Leishmania (136, 137), EBV (138), HIV (101, 139, 140) and hepatitis B (HBV) (141, 142).

High levels of IL-10 have been documented in systemic lupus erythematosus (143, 144), multiple sclerosis (124), rheumatoid arthritis (145) and Sjogren’s syndrome (146), as well as in autoimmune lymphoproliferative syndrome (147), acute ulcerative colitis (148), and Grave’s disease (149).

On the other hand, downregulation of IL-10 expression or defective signaling can also have both a beneficial and a detrimental impact to the host (**Figure 1**). An IL-10 deficiency occurring in the early phase of microbe infection triggers a rapid amplification of the innate and adaptive immune response and facilitates effective clearance of invading pathogens (150). If the deficiency persists, it leads to systemic, exaggerated inflammation and immune-mediated tissue damage and participates to the onset or aggravation of chronic inflammatory diseases and several autoimmune pathologies (74, 79, 151).

As example, IL-10-deficient mice develop colitis (152, 153) and during infection with T. cruzi (154) and T. gondii (135, 155) succumb to an excessive, lethal inflammatory response. IL-10 deficiency has been also demonstrated to aggravate chronic liver and kidney disease, enhancing fibrosis and inflammation (156, 157). IL-10 expression was found lower in psoriatic (158, 159) and asthmatic patients (160) and IL-10 and IL-10R mutations, causing a loss of IL-10 function, were found to be associated with severe inflammatory bowel disease, including Crohn’s disease and ulcerative colitis (52, 161, 162).

With age the functional competence of the immune system declines, a process called immunosenescence and involves the remodeling of innate and adaptive immunity and it is associated with a higher likelihood and severity of several infections (163). Immunosuppressive cells and immunosuppressive cytokines are involved in this process including IL-10 that has been found increased in several studies (164).

The scheme is illustrating the dual immunological activities of IL-10 and the possible beneficial or detrimental impact of this cytokine at high or low levels on human health and disease (**Figure 1**).

## IL-10 in cancer

The role of IL-10 in tumor pathogenesis is currently highly controversial, with some findings showing that IL-10 promotes tumor development and angiogenesis, while others supporting that it inhibits tumor growth and metastasis (115).

This cytokine is considered a master switch from tumor-promoting inflammation to antitumor immunity, thus dysregulation in IL-10 levels can importantly contribute to carcinogenesis and tumor progression (112, 165, 166). Elevated IL-10 level exerts tumor-promoting effects by stimulating tumor cell growth and proliferation via STAT3 activation, by inhibiting apoptosis and by allowing immune surveillance escape through inhibition of DC function, downregulation of human leukocyte antigen (HLA) class I molecules on tumor cell surface, recruitment of Treg, suppression of NK cells cytotoxic activity and impaired activation of Th1 CD4+ and cytotoxic T cells (167–172). Increased IL-10 expression in primary tumor cells and tumor-associated macrophages has been proposed as a predictor of cancer stage progression and metastatic potential development (87, 173, 174). Moreover, IL-10 circulating levels were found to be elevated in serum of various cancer patients, often accompanied by the increase of other inflammatory markers, and correlated with a poor prognosis (140, 175–183).

On the other hand, IL-10 mediates important tumor-inhibiting activities by recruiting and stimulating cytotoxic CD8+ T cells and NK cells in the tumor microenvironment, by promoting lymphocyte and antibody-dependent immune memory, by abrogating inflammatory M1 macrophage-Th17 T cells axis, by downregulating the synthesis of pro-angiogenic factors and by suppressing local release of pro-inflammatory cytokines that support tumor growth, survival, and invasion (167, 184–188).

## IL-10 as a potential therapeutic opportunity

The increasing knowledge about the essential regulatory role of this cytokine has encouraged investigators to consider IL-10 as a potential therapeutic opportunity (43, 157, 189, 190). Although no therapy has been yet approved to date, systemic administration of recombinant human (rhu) IL-10 has been tested in multiple clinical trials for the treatment of autoimmune and immune-mediated inflammatory diseases (including inflammatory bowel disease, psoriasis, Crohn’s disease, rheumatoid arthritis, ulcerative colitis, pancreatitis), tissue damage, and chronic infectious diseases (such as chronic hepatitis C), due to its anti-inflammatory, wound repairing and anti-fibrotic functions, respectively (191–194).

Early phase I and II studies showed a trend toward favorable responses of systemically administered IL-10 in psoriasis and Crohn’s disease patients, but larger studies revealed only a slight clinical benefit, due to the double anti- and pro-inflammatory properties of this cytokine (43, 195). Results obtained in a mouse model of human multiple sclerosis suggested that inducing local expression of IL-10 in the site of inflammation has the potential to prevent autoimmune inflammatory process in the central nervous system (43, 195). Induction of a homogeneous population of IL-10-producing CD4 T cells by a combination of immunosuppressive drugs (vitamin D3 and dexamethasone) may represent a promising therapeutic strategy for the treatment of autoimmune and inflammatory diseases (43, 195). From the other side, the use of anti-IL-10R mAbs potentiate the Th1 response and may be useful for the development of effective vaccines and to enhance appropriate immune responses against chronic pathogens (43, 195).

Moreover, given the double tumor-promoting and tumor-repressing IL-10 action, both blocking and systemic administration of IL-10 have been explored as potential strategies for cancer immunotherapy.

Yet, IL-10’s biologically active form is an unstable homodimer with a short half-life and low *in vivo* stability. This represents a significant drawback of using IL-10 in therapeutic application (51). IL-10’s therapeutic potential can be increased by pegylation, a modification of IL-10 by covalent conjugation with non-toxic polymer polyethylene glycol (PEG), that increase the half-life of a protein following administration (112, 196). It was observed that systemic administration of PEGylated human IL-10 (pegilodecakin) promotes infiltration, activation and intratumor expansion of tumor-specific CD8+ T cells and restores their cytotoxic activity, resulting in enhanced granzyme B and IFN-γ production in CD8+ cells, enhanced intratumor antigen presentation and induction of anti- tumor immune response with evidence of clinical benefits in different advanced solid tumors, such as renal cell carcinoma and uveal melanoma (112, 191, 197, 198). On the other hand, cancer immunization with simultaneous IL-10 signaling blockade, using IL-10R monoclonal antibodies, soluble IL-10R, peptide-based IL-10R antagonists, or oligonucleotides, raised tumor immune response with evidence of clinical benefits in different advanced solid tumors, such as renal cell carcinoma and uveal melanoma (112, 191, 197, 198), and enhances CD8+ T cell response and potentiates vaccine-induced tumor regression (189).

Concomitant blockade of IL-10 and PD-1 immune checkpoint in a mouse model of lymphocytic choriomeningitis virus (LCMV) increases the efficacy in restoring antiviral T cell responses and controlling persistent viral infection (199). Combined treatment with IL-10 and PD-1 blockers enhances the expansion and function of tumor-infiltrating CD8+ T cells, resulting in a synergistic anti-tumor effect in metastatic melanoma and ovarian cancer (189). Recently, therapy with PEGylated-IL-10 and anti-PD-1 monoclonal antibody (pembrolizumab or nivolumab) has shown encouraging results in a phase 1b clinical trial conducted on advanced refractory renal cell carcinoma and non-small-cell carcinoma patients (200).

## Potential role of IL-10 in COVID-19

Chronic viral infections are another field in which IL-10 appears as an intriguing therapeutic challenge. Studies have demonstrated that blockade of IL-10 is able to restore T cell antiviral activity, enhance vaccine efficacy and promote immune-mediated eradication of viral persistence in case of cytomegalovirus, lymphocytic choriomeningitis virus, HIV, and HCV infections (98, 201, 202).

ARDS is the most common complication of Coronavirus disease 2019, affecting approximately 75% of COVID-19 patients in intensive care units (ICU), and a leading cause of COVID-19-releated death (203). It is a progressive respiratory insufficiency, defined by a plethora of symptoms including severe hypoxemia, increased respiratory work, pulmonary embolism, microvascular thrombosis, diffuse alveolar damage with alveolar cell death, edema, fibrosis and inflammatory cells infiltrate into the lung interstitium and alveoli, which can evolve in systemic tissue damage and multiple organ failure and eventually results in a fatal outcome (204–207).

SARS-CoV-2 virus enters in the target cells through the binding of its viral spike protein (S) to the host angiotensin converting enzyme 2 (ACE2), which is present in different human organs (oronasal and nasopharyngeal mucosa, lung, stomach, colon, skin, lymph nodes, liver, kidney, brain) and mainly expressed on lung alveolar epithelial cell type II, enterocytes of the small intestine and vascular endothelium (207, 208). Even though the exact sequelae of mechanisms leading to COVID-19-mediated lung damage are still being delineated, it is widely accepted that cytokine storm plays a prominent role (15, 16, 209–211). Alveolar epithelial cells, alveolar macrophages and dendritic cells function as sensor cells of the respiratory mucosa and, upon SARS-CoV-2 infection, give rise to immune response with a first huge release of early pro-inflammatory cytokines (including IFN-α, IFN-γ, IL-1β, IL-2, IL-6, IL-12, IL-18, IL-23, TNF-α) and chemokines (such as CCL2, CCL3, CCL5, CXCL8, CXCL9, CXCL10) that activate resident lymphocytes and stimulate recruitment of effector cells (210, 212). Protracted cytokine and chemokine overproduction causes massive recruitment of neutrophils, eosinophils and NK cells in the pulmonary parenchyma. Once there, neutrophils secrete free radicals, myeloperoxidase and other proteases, eosinophils release major basic proteins and cationic proteins, while NK cells liberate granzymes and perforins. All these substances exert cytotoxic effects and lead to alveolar injury. Resident macrophages polarize to M1 phenotype and, in concert with infiltrating DCs, produce nitric oxide and additional pro-inflammatory molecules, such as TNF-α, which induce alveolar cell death and further contribute to pulmonary endothelium damage. Cytotoxic T cells, in turn, migrate to lungs upon activation by DCs, and also participate in the killing of infected cells (210, 213).

The host immune response, active in the first phase, can positively affect infection resolution, suppressing viral replication and leading to complete pathogen elimination and homeostasis restoration. However, excessive inflammation is deleterious and triggers a vicious circle that is self-sustaining of the ongoing hyper-inflammatory state. The resulting dysregulated cascade of cytokine first causes the disruption of the lung epithelial barrier and then, traveling through the bloodstream, can further amplify the cytokine storm, giving rise to systemic inflammation and potentially damaging multiple organs throughout the body (209).

Several pro-inflammatory molecules can variably participate to the cytokine storm driving ARDS in COVID-19, as demonstrated by different clinical studies reporting higher circulating levels of one or more immunoactive molecules in patients with severe form of COVID-19, including IL-1β, IL-2, IL-6, IL-7, IL-8, IL-17, TNF-α, IFN-γ, granulocyte-macrophage colony-stimulating factor (GM-CSF), CXCL10, CCL2, CCL7, CCL3, CCL4, and C-reactive protein (CRP) (209, 214–220). Overweight and obesity are considered a main risk factor for severe symptoms of COVID-19 and increased mortality. This can be explained by the finding that obese patients have altered NK cell polarization, increased levels of pro-inflammatory cytokines, such as IL-6 and TNF-α, and hyperactivation of mTOR pathway (221), besides cardiovascular co-morbidity.

The uncontrolled overproduction of pro-inflammatory chemokine/cytokines observed in SARS-CoV-2 infection is a clinical characteristic in common with that previously seen in SARS-CoV and MERS-CoV infections (211, 212). Although this pathogenic process is shared between COVID-19 and the other beta-coronavirus infections, the massive increase of IL-10 levels in patients with severe forms of the illness is a clinical feature that uniquely distinguishes SARS-CoV-2 infections (211, 222).

A large increase in the proportion of IL-10-secreting regulatory T cells has been found in peripheral blood of patients with severe COVID-19, compared to those with mild-to-moderate cases and healthy individuals (223). Several studies have also reported that circulating levels of IL-10 are significantly elevated in severe cases of COVID-19, especially in patients admitted to the ICU compared to those not requiring ICU care (224, 225) and continued to increase after admission (45, 116). In addition, elevated IL-10 levels are seen in patients developing ARDS, respiratory failure and extrapulmonary dysfunction like disseminated intravascular coagulation (116, 207) and severe acute kidney injury (46, 226), as well as a reduced patient survival (116, 227, 228).

Higher IL-10 levels have been positively correlated with increased exhaustion markers PD-1 and TIM-3 expression on T cells and lower total number of CD4+ and CD8+ T cells (225). In patients with severe forms of COVID-19 also a strong relationship between early overexpression of IL-10 and increased serum concentrations of IL-1 receptor antagonist (IL-1RA) and other proinflammatory molecules, including IL-6, IL-8 and C-reactive protein (116, 215, 228–230) was observed. Numerous clinical studies have also revealed that elevated amounts of IL-10 in the serum of COVID-19 patients, alone or with IL-6, IL-12 or IL-1RA, may accurately predict progression to more severe form of disease and increased mortality (46, 215, 229–234).

Taken together, these evidence have robustly supported the great potentiality of monitoring circulating levels of IL-10 in COVID-19 patients as reliable biomarker to rapidly predict the disease course at the first stages of infection, to early recognize patients with higher risk of developing detrimental complications (231) and to accurately determine the most suitable therapeutic options and the right time of treatment administration (228, 234).

Alternative potential scenarios were proposed to explain the clinical meaning of the increase in IL-10 levels in serum of COVID-19 patients occurring within a few days from infection. IL-10 level significantly increases one week after symptoms onset following the massive release of pro-inflammatory cytokines that occurred in the preceding days. A study showed statistically significant differences of IL-10 serum levels in the non-severe and severe groups on days 0, 3, and 6. The median concentration of IL-10 on days 3 and 6 was increased in both the non-severe and severe groups compared to day 0 (235). IL-10 is generated after acute infection and could have the ability to block the expression and production of numerous proinflammatory cytokines, preventing the development of excessive or chronic immune activation.

**Figure 2:**
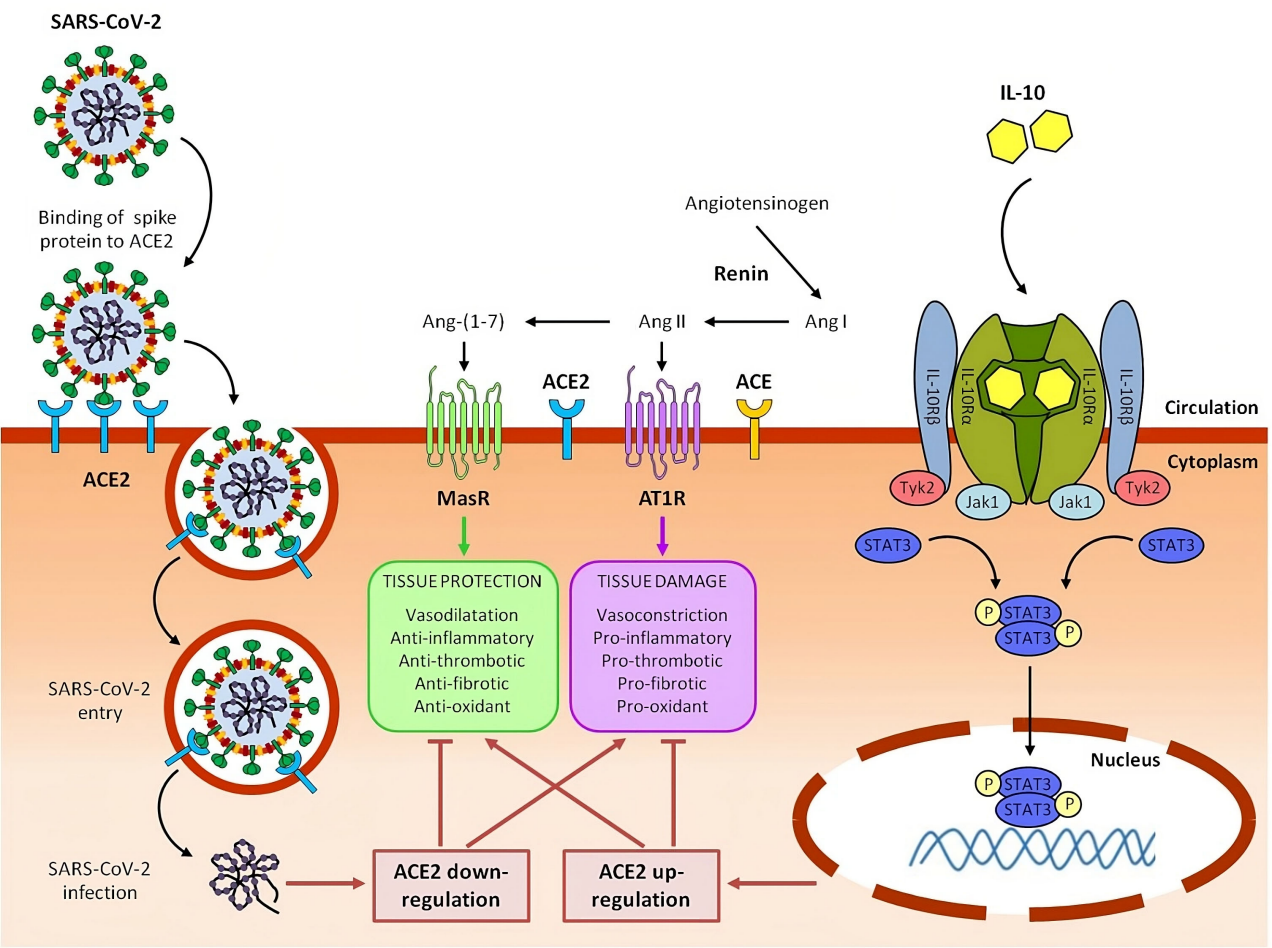
Potential role of IL-10 in counteracting ACE2 downregulation, rebalancing RAAS system and mitigating tissue damage caused by SARS-CoV-2 in severe COVID-19 and Long COVID syndrome.

The first possible explanation of the COVID associated data, suggests in fact that IL-10, in the context of ongoing inflammation induced by COVID-19, behaves in a canonical way as an anti-inflammatory and immunosuppressive cytokine. High circulating levels of IL-10 could be interpreted like an endogenous danger signal, activated as a negative feedback mechanism in response to the dramatic increase of pro-inflammatory mediators and the related alveolar endothelial cell damage. Therefore, IL-10 acts as an attempt of the host organism to protect itself from the deleterious effects of an excessive inflammatory reaction, preventing further progression of tissue damage (211, 236) (**Figure 2**).

In the renin-angiotensin system (RAAS), renin converts angiotensinogen to angiotensin I (Ang I), which is in turn converted to angiotensin II (Ang II) by angiotensin-converting enzyme (ACE). Ang II, acting through Ang II type 1 receptor (AT1R), promotes inflammation, fibrosis, vasoconstriction, thrombosis and oxidative stress, ultimately resulting in tissue injury. Detrimental effects of Ang II are counterbalanced by Angiotensin-converting enzyme 2 (ACE2), which converts Ang II to angiotensin 1-7 (Ang-(1–7)). Ang-(1-7), signaling through the Mas receptor (MasR), inhibits inflammation and mediates tissue protection.

SARS-CoV-2 infects host cells by binding its viral spike protein to the receptor ACE2. Following this binding, SARS-CoV-2 is internalized by endocytosis and ACE2 expressed on the cell plasma membrane is downregulated. Reduction of ACE2 leads to RAAS imbalance with an increase of the ACE/Ang II/AT1R axis and a parallel decrease of the ACE2/Ang-(1–7)/Mas-R axis, contributing to hyperinflammation and tissue damage of COVID-19 and Long COVID syndrome.

Circulating interleukin 10 (IL-10) binds as a homodimer to tetrameric IL-10 receptor (IL-10R) complex and induce the downstream activation of Janus kinase 1 (Jak1) and tyrosine kinase 2 (Tyk2) and the subsequent phosphorylation of signal transducer and activator of transcription 3 (STAT3). Phospho-STAT3 (p-STAT3) homodimers translocate into the nucleus, where they directly bind to specific sequences and regulate the transcription of its target genes, including anti-inflammatory genes and ACE2. Upregulating ACE2, IL-10 can help to restore RAAS balance, with a reduction of ACE/Ang II/AT1R axis and an increase of ACE2/Ang-(1–7)/MasR axis, resulting in beneficial effects on COVID-19 and post-COVID-19 symptoms. We have previously reported that, in lung-derived and endothelial cell lines, IL-10 administration increased the expression level of SARS-CoV-2 receptor, ACE2 a potent anti-inflammatory molecule (237) (**Figure 2**).

Blood level of IL-10 is low following SARS-CoV-2 infection during the innate immune response phase and starts to be significantly increased around 3 days/one week following the massive release of inflammatory cytokines (TNF-α, IL-1, IL-6) and symptoms onset. IL-10 raises after acute disease. During the convalescent phase, IL-10 levels slowly decrease along with the symptoms in about 2-3 weeks.

Thus, IL-10 could behave as a counter-regulator of the local endothelial inflammation as well as the systemic inflammatory process, by enhancing ACE2 expression (237). In an ex-vivo study on peripheral-blood immune cells, we have also recently demonstrated that IL-10 treatment decreased the IFN-γ specific response to spike stimulation, decreased the release of numerous pro-inflammatory cytokines, chemokines and growth factors, reduced the frequency of IFN-γ producing CD4, CD8 and NK cells and cell activation (evaluated by HLA-DR expression), in both COVID-19 patients and NO COVID-19 vaccinated subjects (45). Our study further confirmed the view of an immunomodulatory role of IL-10 in the SARS-CoV-2 specific inflammatory response and highlights the therapeutic potential of the administration of rhu IL-10 to treat ARDS in COVID-19 patients, as already investigated for solid tumors and various autoimmune and inflammatory diseases (45).

**Figure 3:**
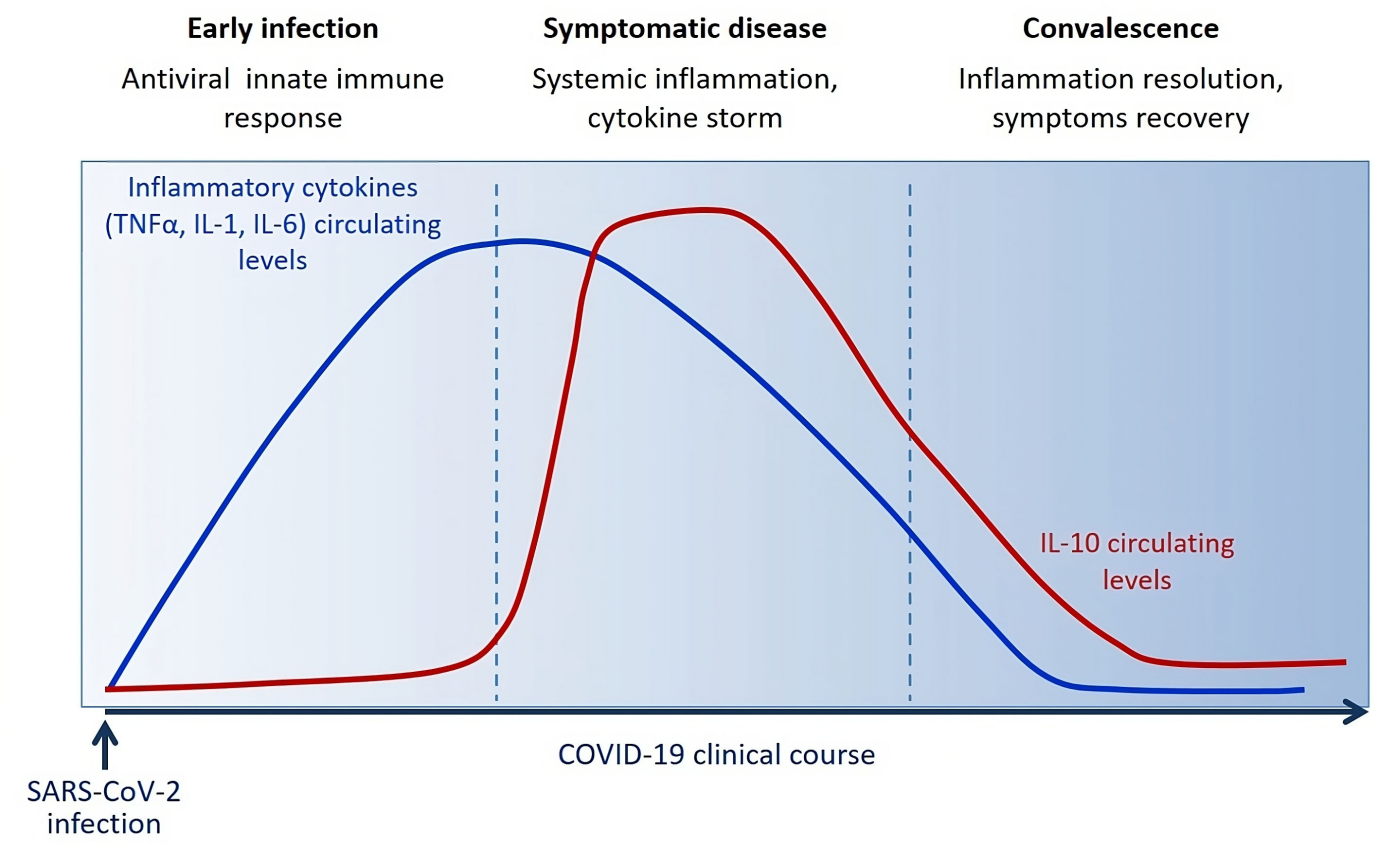
Circulating levels of IL-10 dynamically change during the clinical course of COVID-19 reflecting host immune/inflammatory state.

Several clinical studies have described a huge increase in IL-10 early after few days from infection, after the concomitant increase of various other pro-inflammatory cytokines (such as TNF-α, IL-6, IL-1) (**Figure 3**), as distinctive trait of the hyperinflammatory state developed upon SARS-CoV-2 infection (238). It has also been observed a strong association between IL-10 levels and COVID-19 severity and outcome, suggesting that IL-10 fails to adequately turn off the inflammation. A plausible explanation for this emerging evidence concerns the potential resistance or hypo-responsiveness of activated immune cells to the immunosuppressive action of IL-10, resulting in uncontrolled and self-sustained release of pro-inflammatory cytokine into circulation (239). The occurrence of this situation has already been demonstrated *in vitro* in high-glucose conditions and *in vivo* in patients diagnosed with type 2 diabetes and has been attributed to defective STAT3 activation (151). The impaired IL-10 response in presence of high glucose can justify the increased frequency of mortality and severe complication in COVID-19 patients with diabetes or hyperglycemia and the better outcomes associated with improved glycemic control (236, 240). Therefore, it is reasonable to speculate that pharmacological strategies able to overcome resistance and/or restore responsiveness to IL-10, as happens by the treatment with a small molecule agonist of SHIP1 (Src homology-2 containing inositol-5’-phosphatase 1) in macrophages under hyperglycemia (151), could give a valid therapeutic opportunity to reduce the overwhelmed inflammation in patients with severe COVID-19, especially those with diabetes.

In severe COVID-19 cases a drastic early rise in IL-10 was observed, an effect that represents a paradoxical role of this cytokine in its classical anti-inflammatory role. This observation gives a convincing justification for the increased IL-10 levels in the presence of systemic inflammation such as COVID-19 condition, as well as previously observed in cancer and immunity (115, 241) (**Figure 2**). This can be explained with IL-10 “resistance”, as reported by Islam et al., 2021, hypothesis that requires further investigation (239).

Different studies have previously revealed hyper-activation and expansion of CD8+ T cells, enhanced production of IFN-γ and peripheral increase of various pro-inflammatory and immune-activating mediators following recombinant IL-10 administration in healthy subjects with LPS-induced endotoxemia and in patients with Crohn’s disease and some cancers. Most cytokines/chemokines are reported as upregulated in these studies (IL-2Rα, IL-4, IL-7, IL-18, IFN-γ, GM-CSF, TNF-α, CXCL10 and CXCL9) supporting the potential immunostimulatory action of this cytokine in severe COVID-19 (45). In addition, elevated levels of LPS, a potent stimulators of IL-10 secretion by macrophages, were observed in plasma of patients with severe COVID-19 (239). In this scenario, stimulation of IL-10 signaling with PEG-IL-10 or other IL-10 stimulation might result in clinical benefit for patients with severe COVID-19.

It is also possible that IL-10 plays a double role in COVID-19, depending on the timing of the secretion: after few days from infection, IL-10 produced in the lungs, after viral infection, may work as a negative feedback mechanism started by an increased proinflammatory mediators release and aimed at counter-modulating inflammation and restoring tissue homeostasis. However, in the later phases, IL-10 production becomes continuous and elevated and may act as an immune stimulating factor that promotes a further release of proinflammatory cytokines/chemokines, hyperactivates cytotoxic effector CD8+ T cells and amplifies systemic inflammation, leading to disease exacerbation (211).

## Potential role of IL-10 in post-COVID-19 syndrome

One of the main problems aggravating the sanitary emergency due to SARS-CoV-2 pandemic, is the management of the estimated 10% of patients who do not undergo a complete recovery but manifest persistent post-COVID-19 symptoms for up to 12 or more weeks after initial infection (24, 242).

The plethora of post-COVID-19 symptoms is highly heterogeneous and comprises variable degrees of severity: physical symptoms as fever, fatigue, respiratory symptoms, as dyspnea, breathlessness and coughing, painful symptoms, as myalgia, arthralgia, headache and chest pain, neurological symptoms, as anosmia, dysgeusia, difficulty concentrating and sleeping, psychological symptoms, as depression, anxiety, poor memory and concentration, cardiovascular symptoms, as tachycardia and coagulation dysfunction, and gastrointestinal symptoms. These multi-organ symptoms can occur as a result of organ damage following severe COVID-19 or arise *de novo* after mild infection without evidence of organ injury (24, 25, 28, 242, 243).

The clinical spectrum of post-COVID-19 symptoms was classified, by Fernández-de-Las-Peñas and colleagues, into three different phases based on a temporal criterion: acute post-COVID symptoms (from 4-5 to 12 weeks after infection), long post-COVID symptoms (from 12 to 24 weeks), and persistent post-COVID symptoms (lasting more than 24 weeks) (244). The precise mechanisms responsible for post-COVID-19 pathology remains still unclear, but different causative factors have been proposed to contribute to the various clinical sequelae observed in patients (26, 245, 246). Firstly, SARS-CoV-2, by infecting and replicating into ACE2 expressing cells, can exert a direct viral toxicity and cause diffuse endothelial cell damage (247, 248). SARS-CoV-2-mediated endothelial damage, by recruiting and activating immune cells and promoting pro-inflammatory and pro-thrombotic mediators release, can trigger subsequent endothelial inflammation leading to thrombosis and vascular damage (207, 247). Viral entry into cells mediates the downregulation of ACE2 and its consequent failure to convert the angiotensin II into angiotensin 1-7, resulting in the accumulation of angiotensin II and overstimulation of RAAS that ultimately causes hypertension, electrolyte unbalancing, lung fibrosis and inflammation, vasculitis, thromboembolism and intravascular disseminated coagulation (207, 249). In addition, SARS-CoV-2 impairs the mitochondrial antioxidant function, resulting in increased reactive oxygen species (ROS) release, oxidative stress and oxidative damage, which lead to tissue damage, thrombosis, and red blood cell dysfunction (250–252). The other fundamental mechanism contributing to the pathological process is thought to be host’s immune response dysregulation. SARS-CoV-2 dissemination can trigger a massive cell activation to induce an anti-viral immune response with an exaggerated and continual production of inflammatory cytokines, that lead to alveolar edema, hypoxia, thrombosis, tissue damage, and can ultimately results in systemic inflammatory response involving the whole organism and causing a multi-organ injury (207, 247, 248).

Given the critical role played by IL-10 in the promotion of tissue repair and resolution of inflammation it is possible that this cytokine could have a useful impact in recovering physiological homeostasis and ending the post-COVID-19 symptoms. Blood level of IL-10 is significantly increased in the first week following the symptoms’ onset in patients who developed severe COVID-19. Moreover, higher serum levels of IL-10 were found in individuals who did not experience sequelae after acute infection compared to subjects with post-COVID-19. This supports the idea that elevated levels of IL-10 in the post-COVID-19 period, allow a more efficient resolution of the immunopathological process, by improving anti-inflammatory response (253).

Among the symptoms of post-COVID-19 on which IL-10 could have a beneficial effect there is the olfactory and gustatory dysfunction (OD/GD), a distinctive sign of acute COVID-19 and one of the most frequent long-lasting complications in post-COVID-19 (254). Locatello and colleagues have reported that elevated serum concentration of IL-10 on hospitalization, compared to increased levels of other cytokines or presence of clinical comorbidities, is the only significantly parameter associated with 30-day taste recovery (255). Luporini et al. has reported higher IL-6 and IL-10 levels in serum of adults over 65 with COVID-19, associated with disease severity and a higher comorbidity index (222). This evidence further supports an involvement of inflammatory process in COVID-19-associated chemosensory dysfunction and suggests a role for IL-10 as reliable predictor of OD/GD course as well as potential pharmacological strategy to reach a successful recovery in post-COVID-19 patients.

Pain is another post-COVID-associated pathological manifestation in which IL-10 may have a clinical utility. In particular, joint, muscle and chest pain represent one of the most frequently reported persistent symptoms after the resolution of acute COVID-19 infection and Bussmann et al. (256) have observed a strong inverse correlation between circulating levels of IL-10 and pain intensity in COVID-19 patients (256). This evidence suggests an analgesic function for IL-10 in the context of post-COVID-19 and proposes that this cytokine can significantly improve the patient’s quality of life, resolving the chronic pain debilitating condition (256).

Cardiovascular and respiratory symptoms are other persistent clinical signs, among those commonly affecting post-COVID-19 patients, which can be positively influenced by IL-10. Virus-mediated downregulation of ACE2, the counter-regulator of ACE, may cause dysregulation of the renin-angiotensin-aldosterone system (RAAS), resulting in a worsening of cardiovascular and respiratory condition (257). Absence of angiotensin-converting enzyme inhibitors (ACEI)/angiotensin II receptor blockers (ARBs) therapy were the main prognostic indicators of in-hospital mortality (258). As reported before, IL-10 could increase ACE2 expression in lung and endothelial cells (237). Therefore IL-10, by restoring RAAS balance, can importantly contribute to normalization of electrolyte levels and blood pressure, containment of pulmonary inflammation and fibrosis, resolution of vasculitis, thrombosis, and hyper-coagulation (207).

Although the exact function played by IL-10 in COVID-19 has not yet been fully defined, due to the multifaceted actions exerted on inflammation, IL-10 has been increasingly proposed as critical contributor during the kinetics of cytokine storm, which is considered a main responsible for the development and progression of ARDS in COVID-19 patients and a keystone factor in influencing disease morbidity and mortality (211, 212, 259).

In fact, IL-10 can also have a beneficial impact in mitigating or even suppressing the continuative systemic inflammation typically associated to post-COVID-19 syndrome. In our recent research, we have demonstrated that exogenous delivery of IL-10 to whole-blood cells downregulates SARS-CoV-2 induced exacerbated inflammatory response, by reducing several pro-inflammatory mediators correlated with COVID-19 severity and by decreasing frequency and activation of IFN-γ producing CD4, CD8 T cells and NK cells (45).

It is also possible that exogenous IL-10 plays a useful therapeutic role in counteracting neurological symptoms observed in post-COVID-19. In this regard, Trandem and colleagues (260) have shown the protective effects of elevated IL-10 levels in mice infected with a neurotropic coronavirus (260). High IL-10 production, occurring during the early phase of viral encephalitis, leads to decreased microglia activation, immune cells infiltration and proinflammatory factors release and an increased regulatory T cell rate in the site of infection. The immunomodulating actions of IL-10 were long-time lasting and manifested during the resolution phase of the infection, resulting in decreased demyelination and improved survival (260).

## Natural bioactive compounds influencing IL-10 production

The therapeutic role of bioactive compounds obtained from plants in the treatment of human diseases has been extensively acknowledged (261–265).

Considering the potential wide-ranging impact that Il-10 could have on complications associated to post-COVID-19 syndrome, and in other diseases, such as cancer, it is of interest to study natural bioactive compounds, able to increase IL-10 expression and enhance its action, which could represent a useful therapeutic strategy. In **Table 1** we report bioactive compounds, derived from natural sources, that influence IL-10 production. Among these compounds, the polyphenol curcumin is endowed with numerous beneficial properties, including antioxidant, anti-inflammatory, anti-nociceptive, anti-fatigue and anti-fibrotic effects, by increasing the expression, production, and activity of IL-10 (266–268). Administration of nano-curcumin has been reported to provide anti-viral action and to downregulate expression and secretion of the inflammatory cytokines IL-1β and IL-6 in COVID-19 patients (292, 293). The polyphenol 6-gingerol can upregulate IL-10 production and possesses useful therapeutic effects, comprising antioxidant, anti-inflammatory, immunomodulatory, analgesic, antipyretic and anti-SARS-CoV-2 activity (268, 269). The green tea polyphenol epigallocatechin-3-gallate (EGCG) induces Treg by increasing Foxp3 and IL-10 expression in CD4 T cells (270, 271), while acteoside, a phenolic glycoside, can promote B cell-derived IL-10 production, ameliorating inflammatory process (272). The natural dietary polyphenols kaempferol and resveratrol, with known anti-inflammatory, antioxidant, antimicrobic and disease‐protective activities, stimulate IL-10 production and inhibit inflammatory cytokine secretion (273). A similar effect on oxidative stress, inflammation and IL-10 level was obtained with a diet enriched in high-polyphenols containing Extra Virgin Olive Oil (EVOO) (275). The flavonoids quercetin (276), naringin (277), apigenin (278, 279), luteolin (280), present in different vegetables and fruits, and the alkaloid piperin (281) and S-1-Propenylcysteine (286), are other examples of natural compounds able to increase IL-10 levels and exert antioxidant, anti-inflammatory, immunomodulatory, anti-cancer and antimicrobial properties (268, 274, 280, 294).

**Table 1:** Bioactive compounds, derived from natural sources, influencing IL-10 production.

Natural compounds	
Plant-derived compounds	References
Curcumin	(266–268)
6-gingerol	(268, 269)
Epigallocatechin-3-gallate (EGCG)	(270, 271)
Acetoside	(272)
Kaempferol and Resveratrol	(273, 274)
Extra Virgin Olive Oil (EVOO)	(275)
Quercetin	(276)
Naringin	(277)
Apigenin	(278, 279)
Luteolin	(280)
Piperine	(281)
Lupeol	(282, 283)
Arctigenin	(284)
Andrographolide	(285)
S−1−Propenylcysteine	(286)
Marine-derived compounds	
Marennine	(287)
Ulvan	(288)
Asperlin	(289)
Diatoxanthin	(290)
Astaxanthin	(291)

Evidence emerging from literature and clinical trials suggests that dietary-derived polyphenols could represent a helpful supplement in COVID-19 therapy, by contrasting viral load, suppressing inflammation, promoting ACE2/Ang- (1–7)/MasR axis, protecting organs from damage, preventing complications and reducing illness severity (295).

IL-10 production has been demonstrated to be significantly increased in macrophages M1 treated with lupeol (pentacyclic triterpene Lup-20(29)-en-3-ol), a secondary metabolite which is primarily present in fruit plants (282, 283). Similar results were observed by Hyam et al. in 2,4,6-trinitrobenzene sulfonic acid (TNBS) induced colitis model treated with arctigenin, present in Arctium lappa (burdock plant) seeds (284). Moreover, it has been observed that the treatment with andrographolide, a bioactive compound present in the plant known as Andrographis paniculate, increase IL-10 expression in LPS stimulated primary glial culture (285).

In addition, marine environment represents a rich reservoir of immunoactive molecules, mainly concentrated in photosynthetic organisms such as microalgae, which have been recently considered bioactive cell factories for human health benefits (296). Marine organisms have emerged as a source of bio-compounds that could be used as potential immunomodulatory drugs (297), indeed, different marine compounds show an immunomodulatory function increasing IL-10 levels.

Marennine, a blue pigment produced by Haslea ostrearia, a marine pennate diatom, acts on neuroinflammatory processes, inducing a strong up-regulation of IL-10 genes (287). Ulvan, a sulfated polysaccharide produced from a green marine algae Ulva Ohnoi, showed a mild immunomodulatory function increasing IL-10 levels (288). Zhou et al. reported that asperlin, derived from the marine fungus Aspergillus versicolor shown beneficial properties again atherosclerosis, *in vitro* and *in vivo*, due to the increase of protective cytokines (IL-10 and IL-4) (289).

In this scenario marine microalgae are emerging as rich sources of a wide range of bioactive metabolites with anti-antioxidant and anti-inflammatory properties that can serve as potential new therapeutic agents to treat or prevent the severe symptoms of COVID-19, possibly by enhancing IL-10 levels (298, 299). Marine sulfated polysaccharides, isolated from different algae, have shown anticoagulant and immunomodulatory activities as well as potent antiviral properties, both by stimulating innate immune system and mucosal barrier defense against the virus and by preventing viral entry, replication and proliferation (299–301). We have recently demonstrated that diatoxanthin, a carotenoid derived by marine diatoms, significantly upregulates IL-10 production, increases ACE2 activity, exerts an immunomodulant effect by up-regulating antiviral defense genes and by strongly inhibiting spike-induced inflammatory response in a lung cell line. Diatoxanthin, exclusively found in marine environment, decreases the release of pro-inflammatory mediators responsible for cytokines storm in SARS-COV2 disease, supporting the therapeutic potential of marine-derived bioactive compounds against COVID-19 (290).

Among bioactive molecules derived from marine microalgae, there are some polyphenols also known to exert antiviral activities, such as the two flavonoids kaempferol and apigenin that are natural down-regulators of ACE. Apigenin upregulates the expression of the ACE2 enzyme in kidneys inducing a blood pressure decrease effect, potentially effective for viral disease control (e.g. COVID-19). In addition, apigenin and kaempferol inhibit RAAS, which participates in virus entry into lung cells in the case of coronavirus infection (302).

Astaxanthin is another carotenoid of microalgal origin that provides a rationale to be investigated as a potential beneficial additive in COVID-19 therapeutics. Astaxanthin has potent antioxidant, anti-inflammatory, and immunomodulatory effects, can increase IL-10 secretion and exert a protective role against cytokine storm and hyper-inflammation, preventing severe complications (291, 303).

## Conclusions

IL-10 is a critical mediator of host innate and adaptive immunity. It has a multifaceted nature in stimulating or inhibiting crucial immune pathways. IL-10 as an immune modulator can decrease detrimental inflammation, inhibit cancer progression, and curbing disease conditions. It has come to reviewed attention due to its important level changes in certain COVID-19 patients.

COVID-19 and its long-lasting complications resulting in post-COVID-19 syndrome have been and continue to be a global emergency and a severe challenge to healthcare systems around the world. It has been observed that the trigger of cytokine storm and the following hyper-inflammatory state are key causative factors for the development of severe symptoms and complications. Although different cytokines have been found as deregulated in COVID-19 patients, IL-10, due to its multifaceted role in modulating inflammation, appears as one of the most intriguing. Present findings support the potential of this cytokine as reliable predictor of the severity and the outcome in COVID-19 patients, as a possible danger factor and as novel strategy to counteract hyperinflammation, not only in the acute SARS-CoV-2 infection phase but also in the post-infection period.

Further studies are needed to elucidate whether exogenous administration of IL-10 or molecules able to act as adjuvant for the activation of anti-inflammatory IL-10 signaling may be beneficial for ameliorating COVID-19 and post-COVID-19 symptoms. More investment in investigation of IL-10 pathway therapy could be useful in cancer and other chronic diseases. Natural molecules have also been revealed to be modulators of this pivotal cytokine.

## Author contributions

AA and DG conceptualization, EA, VC writing original draft, DN, AA, LC, GD, CS review and editing. All authors contributed to the article and approved the submitted version.
